# Ischemic Colitis after Cardiac Surgery: Can We Foresee the Threat?

**DOI:** 10.1371/journal.pone.0167601

**Published:** 2016-12-15

**Authors:** Rawa Arif, Mina Farag, Marcin Zaradzki, Christoph Reissfelder, Frank Pianka, Thomas Bruckner, Jamila Kremer, Maximilian Franz, Arjang Ruhparwar, Gabor Szabo, Carsten J. Beller, Matthias Karck, Klaus Kallenbach, Alexander Weymann

**Affiliations:** 1 Department of Cardiac Surgery, Heart and Marfan Center—University of Heidelberg, Heidelberg, Germany; 2 Department of Visceral, Thoracic and Vascular Surgery, University Hospital Carl Gustav Carus, Technical University Dresden, Fetscherstr. Dresden, Germany; 3 Department of General, Visceral and Transplantation Surgery, University Hospital Heidelberg, Heidelberg, Germany; 4 Institute of Medical Biometry and Informatics, University of Heidelberg, Heidelberg, Germany; 5 Department of Cardiac Surgery, HaerzZenter-INCCI, rue Ernest-Barblé, Luxembourg, Luxembourg; Azienda Ospedaliero Universitaria Careggi, ITALY

## Abstract

**Introduction:**

Ischemic colitis (IC) remains a great threat after cardiac surgery with use of extracorporeal circulation. We aimed to identify predictive risk factors and influence of early catecholamine therapy for this disease.

**Methods:**

We prospectively collected and analyzed data of 224 patients, who underwent laparotomy due to IC after initial cardiac surgery with use of extracorporeal circulation during 2002 and 2014. For further comparability 58 patients were identified, who underwent bypass surgery, aortic valve replacement or combination of both. Age ±5 years, sex, BMI ± 5, left ventricular function, peripheral arterial disease, diabetes and urgency status were used for match-pair analysis (1:1) to compare outcome and detect predictive risk factors. Highest catecholamine doses during 1 POD were compared for possible predictive potential.

**Results:**

Patients’ baseline characteristics showed no significant differences. In-hospital mortality of the IC group with a mean age of 71 years (14% female) was significantly higher than the control group with a mean age of 70 (14% female) (67% vs. 16%, p<0.001). Despite significantly longer bypass time in the IC group (133 ± 68 vs. 101 ± 42, p = 0.003), cross-clamp time remained comparable (64 ± 33 vs. 56 ± 25 p = 0.150). The majority of the IC group suffered low-output syndrome (71% vs. 14%, p<0.001) leading to significant higher lactate values within first 24h after operation (55 ± 46 mg/dl vs. 31 ± 30 mg/dl, p = 0.002). Logistic regression revealed elevated lactate values to be significant predictor for colectomy during the postoperative course (HR 1.008, CI 95% 1.003–1.014, p = 0.003). However, Receiver Operating Characteristic Curve calculates a cut-off value for lactate of 22.5 mg/dl (sensitivity 73% and specificity 57%). Furthermore, multivariate analysis showed low-output syndrome (HR 4.301, CI 95% 2.108–8.776, p<0.001) and vasopressin therapy (HR 1.108, CI 95% 1.012–1.213, p = 0.027) significantly influencing necessity of laparotomy.

**Conclusion:**

Patients who undergo laparotomy for IC after initial cardiac surgery have a substantial in-hospital mortality risk. Early postoperative catecholamine levels do not influence the development of an IC except vasopressin. Elevated lactate remains merely a vague predictive risk factor.

## Introduction

Complications of the large intestine occurring in patients after cardiovascular surgery with cardiopulmonary bypass (CPB) are rare adverse events with an incidence of <1% and a poor outcome with a reported mortality of 30–100% [1–4]. Main causes of this complication are mesenteric ischemia or Ogilvie syndrome. Mesenteric ischemia is differentiated into occlusive disease (OMI), which is caused by embolism or thrombosis, and prognostically worse non-occlusive disease (NOMI) [5]. However, the definitive diagnosis is difficult to identify, even after histopathological analysis. Thus, clinical symptoms such as pain, distended or acute abdomen, ileus, fever, bleeding and mostly dilation of the colon accompanied with low output syndrome lead to diagnosis and/or operative intervention. Several studies report high mortality rates caused by this complication and the importance of early diagnosis and intervention [1–7]. Both, the limited understanding of the underlying causes as well as the constant poor outcome without any remarkable improvement must be addressed as an unsolved problem in cardiovascular surgery.

In this single center study, we analyzed perioperative data and asserted predictive risk factors for the development of IC after initial cardiac surgery with use of extracorporeal circulation by matched-pair analysis with emphasis on the influence of early postoperative catecholamine therapy.

## Patients and Methods

### Study Population

We analyzed the retrospective data of all patients who underwent cardiac surgery with CPB in our department between January 2002 and December 2015. Out of ca. 14,000 patients, our database analysis identified 224 patients who underwent colectomy during the postoperative course. We collected the complete records of 58 patients (8 women, mean age: 71 years ± 9 years), who underwent aortic valve replacement (AVR, n = 7), bypass surgery (CABG, n = 40) or the combination of both (n = 11). To optimize the validity of the study and matching we did not include patients undergoing other operative procedures. Some of the patients’ data are part of another cohort and have already been published by one of our authors [7]. After approval of the institutional review board, Ethikkommission der Universität Heidelberg (Ethics committee University of Heidelberg) (S-286/2010) follow-up was obtained through contact with the local population administration office, family doctor or the patient/family directly (only adult patients who are legally competent were included). In accordance with the local ethic committee, the requirement of individual patient consent was waived because of the study’s retrospective design and the data collection from routine care.

### Cardiac Surgical Procedures

All cardiac surgery procedures were performed under use of extracorporeal circulation (ECC). Venous cannulation was executed either bicaval (n = 25) or atrial (n = 33). Activated clotting time (ACT) was determined at 400 seconds by intraoperative heparinization before cannulation. A membrane oxygenator was applied and surgery was performed at different levels of hypothermia (mean: 33°C ± 1.8°C) depending on the surgical procedure. Mean cross-clamp time was 64 minutes (± 33 minutes). Fresh whole blood, erythrocytes, fresh frozen plasma and platelet transfusions were administered if required.

### Abdominal Surgical Procedures

The diagnosis for large intestinal pathology was made by abdominal x-ray, computed tomography with contrast media, endoscopy or exploratory laparoscopy/laparotomy. All patients at our center are treated with purgative medication after the 1^st^ postoperative day (POD). If laxation does not occur by the end of 3^rd^ POD, abdominal diagnostic workup, including abdominal x-ray, computed tomography or endoscopy, accompanied with consultation of our general surgeons are performed. The same procedure is followed, if abdominal symptoms such as pain, distention, rigidity, ileus, fever accompanied with or without elevation of infectious parameters, increase in lactate values or hemodynamic deterioration with need of higher catecholamine levels due to low output syndrome occur. If patients are diagnosed with colon dilation, ischemia or are suspicious for both, laparotomy is performed within 12 hours after decision.

Surgeons of our department of general surgery performed all abdominal surgical procedures. Colon resections in our cohort included right hemicolectomy (n = 9), subtotal colectomy (n = 47), sigmoid-resection (n = 1) and other (n = 1). The decision on the extent of bowel resection was made during laparotomy. The macroscopic ischemic segment of the bowel was resected, and the mucosal wall of the remaining colon was examined. Furthermore, the surgeon always examined whether adequate blood perfusion was present in the remaining mesocolon (arterial bleeding close to the colon) (7). Histopathological analysis of resected segments of the intestine was performed in 95% of the cases.

### Catecholamine doses and Lactate levels

Maximum doses of catecholamine therapy within 12h and 24h postoperatively were collected. Dosage was standardized and given in mcg/mg/min. Catecholamines included Norepinephrine, epinephrine, dobutamine, vasopressin and levosimendan. Catecholamine therapy has usually its highest dosage during the first postoperative day after cardiac surgical procedures and may negatively influence outcome during this early and vulnerable period. Additionally, maximum lactate levels within 12h and 24h postoperatively were also collected and analyzed. Lactate was measured by point-of-care testing and given in mg/dl.

### Matching

The IC cohort was compared to a matched-pair group for statistical analysis in a retrospective manner. Matched patients were also selected during the same time period out of 14,000 patients. Operative procedure, age ± 5 years, sex, BMI ± 5, left ventricular function, peripheral arterial disease, diabetes and urgency status were used for match-pair analysis (1:1). Thus, 58 patients were matched, who did not suffer from IC during the postoperative course and did not underwent laparotomy for other reasons.

### Statistics

Continuous variables are shown as mean ± standard deviation or as median and range, categorical data as percentage. To elaborate differences between both arms preoperative, operative and postoperative data were analyzed by Student’s t-test, Fisher’s exact test and Χ^2^ test. To define perioperative risk factors for the development of IC univariate and multivariate Cox proportional hazards model was applied. For univariate analysis all relevant parameters and variables were tested. Significant univariate factors were analyzed using multivariate regression. Receivers Operating Curve (ROC) was used to determine the predictive efficiency of lactate value, vasopressin and epinephrine therapy within 1^st^ POD. A two-tailed p value less than 0.05 was considered significant. SPSS 22.0 software (SPSS, Inc, Chicago, Ill) was used for all statistical analysis.

## Results

### Preoperative Data

The cohorts of both study arms showed comparable baseline-characteristics with a high presence of NYHA class III and IV (91 vs. 86%; p = 0.398). 29% of the complete cohort underwent emergency operation (p = 1).

Furthermore, hepatic disease (4 patients: 2 idiopathic cirrhosis, 1 cirrhosis due to hemochromatosis, 1 chronic hepatitis B infection) was preoperatively more common in the colectomy group (6.9 vs. 0%; p = 0.035) whereas no difference was found for gastrointestinal disease (8.6 vs. 3.4%; p = 0.231) preoperatively. Only four patients in the non-IC group and five in the colectomy group were dependent on catecholamine therapy preoperatively as shown in Table 1.

**Table 1:** Preoperative Data.

	IC group (n = 58)	Non-IC group (n = 58)	P
female	8 (14)	8 (14)	1
age	70.81 ± 8.9	70.41 ± 8.7	0.809
Emergency operation	17 (30)	17 (30)	1
Logistic euroScore	22.4 ± 19.7	20.9 ± 18.4	0.680
NYHA class III + IV	53 (91)	50 (86)	0.398
Instable angina	4 (6.9)	4 (6.9)	1
Hepatic disease	4 (6.9)	0 (0)	0.035
Gastrointestinal disease	5 (8.6)	2 (3.4)	0.231
Myocardial infarction	31 (53)	32 (55)	1.0
LV-Function			0.369
1 = good	24 (41)	21 (26)	
2 = moderate	15 (26)	22 (38)	
3 = poor	19 (33)	15 (26)	
Dialyses	1 (1.7)	0 (0)	1
Diabetes mellitus	20 (35)	20 (35)	1
Arterial hypertension	54 (93)	50 (86)	0.361
Pulmonary Hypertension	12 (21)	7 (12)	0.316
COPD	16 (28)	13 (22)	0.669
PAD	17 (30)	17 (30)	1
Nicotine	26 (45)	23 (40)	0.707
Creatinine mg/dl	1.44 ± 0.91	1.24 ± 0.62	0.185
Quick %	78 ± 25	89 ± 16	0.052
C-reactive protein mg/l	8 ± 5.8	5.7 ± 12	0.488
Platelets x 109/l	229 ± 94	239 ± 70	0.543
Hemoglobin g/dl	12.5 ± 1.8	12.8 ± 2.9	0.390
Total bilirubin mg/dl	0.66 ± 0.47	0.54 ± 0.24	0.259

COPD, chronic obstructive pulmonary disease; INR, International Normalized Ratio; NYHA, New York Heart Association; PAD–peripheral arterial disease. Data are presented as percentage (n) or mean ± SDM.

### Operative data

The operative data showed some statistical differences between both groups as the IC group had significantly longer operation- (273 ± 122 vs. 203 ± 65 min; p<0.001) and bypass-time (133 ± 68 vs. 101 ± 42 min; p = 0.003), whereas cross-clamp time showed no significant difference (64 ± 33 vs. 56 ± 25; p = 0.150). Blood transfusion was significantly more administered in the IC group (1405 ± 1032 vs. 981 ± 770 ml; p = 0.014) who also required significantly more IABP implantation (31 vs. 8.6%; p = 0.008). Operative outcome data are displayed in Table 2.

**Table 2:** Operation Data.

	IC group (n = 58)	Non-IC group (n = 58)	P
Bypass time, min	133 ± 68	101 ± 42	0.003
Cross-clamp time, min	64 ± 33	56 ± 25	0.150
min. Temperature, °C	33.0 ± 1.8	33.3 ± 1.4	0.217
Red cell concentrate, ml	1405 ± 1032	981 ± 770	0.014
Plasma concentrate, ml	491 ± 810	418 ± 1110	0.689
Platelet concentrate, ml	315 ± 365	225 ± 268	0.135
IABP Implantation	17 (29)	5 (18)	0.008

IABP indicates Intra-aortic Balloon Pump. Data are presented as percentage (n) or mean ± SDM

### Postoperative data

We found numerous significant differences between both groups in the analysis of postoperative data (Table 3). The colectomy group showed a higher maximum Bilirubin value (p = <0.001), suffered more renal insufficiency (p<0.001) and had a significantly longer ventilation time (p<0.001) and more in need of re-thoracotomy (21 vs. 2%; p<0.001). Cardiac low-output syndrome occurred strikingly more in the IC group (71 vs. 14%; p<0.001). During the postoperative course the demand for red cell, plasma and platelet transfusion (p = 0.003, p<0.001, p<0.001) was obviously significantly higher in the IC group.

**Table 3:** Postoperative Data.

	IC group (n = 58)	Non-IC group (n = 58)	P
Invasive ventilation, d	23 ± 22	2 ± 2	<0.001
Low output syndrome	41 (71)	8 (14)	<0.001
Renal failure, d	56 (97)	18 (31)	<0.001
Perioperative MI	5 (9)	4 (7)	1
Delirium, n	17 (8)	15 (7)	0.836
Total bilirubin max., mg/dl	7.37 ± 8.02	1.99 ± 2.40	<0.001
Bleeding, n	10 (17)	4 (7)	0.152
Cerebrovascular event	1 (2)	0 (0)	1

CPR indicates cardiopulmonary resuscitation; MI, myocardial infarction. Data are presented as percentage (n) or mean ± SDM

The maximum doses of catecholamine levels were relatively high in both groups during the initial 24 postoperative hours (Table 4). However, significant differences were only found for vasopressin therapy (1.834 ± 2.977 vs. 0.0 mcg/mg/min, p<0.001) while no patient of the non-IC group required vasopressin therapy (40 vs. 0%; p<0.001). Epinephrine therapy was necessary in 27 (47%) patients who developed IC in the further course and only in 9 (16%) patients who were free of IC (p<0.001). Furthermore, significant differences were found for maximum lactate values between both groups within the first 12 h (p = 0.002) and 24 h (p = 0.002) after initial operation.

**Table 4:** Postoperative catecholamine therapy and lactate values.

	IC group (n = 58)	Non-IC group (n = 58)	P
Norepinephrine 24h max. mcg/mg/min	0.518 ± 2.227	0.645 ± 2.591	0.784
Epinephrine 24h max. mcg/mg/min	0.145 ± 0.228	0.079 ± 0.093	0.099
Dobutamine 24h max. mcg/mg/min	2.40 ± 5.06	2.33 ± 1.37	0.969
Vasopressin 24h max. mcg/mg/min	1.834 ± 2.977	0.0 ± 0.0	<0.001
Levosimendan 24h max. mcg/mg/min	0.004 ± 0.18	0.0 ± 0.0	0.189
Lactate 12h max. (5.7–22) mg/dl	52 ± 47	28 ± 30	0.002
Lactate 24h max. (5.7–22) mg/dl	55 ± 46	31 ± 30	0.002

Data are presented as mean ± SDM

### Abdominal surgery data

Mean time to laparotomy in the IC group was 10.83 ± 11.4 d (range, 2 to 68 days). The etiology of the large intestine disease after intraoperative and histopathological analysis were: 1 patient was diagnosed for occlusive disease (OMI), 57 patients suffered from non-occlusive disease (NOMI) of whom 50 had definite morphological signs of ischemia and 7 dilation of the colon without clear signs of ischemia and were therefore diagnosed with Ogilvie syndrome.

### Risk factors analysis

Univariate logistic Cox regression analysis revealed several variables as predictors for need of colectomy such as maximum lactate level after 24h (HR 1.008, CI 95% 1.003–1.014; p = 0.003). These factors were further asserted by multivariate logistic Cox regression. Only vasopressin doses (HR 1.108; p = 0.027) and most notably low-output syndrome (HR 4.301; p<0.001) remained independent risk factors leading to colectomy during hospital stay (Table 5).

**Table 5:** Univariate and Multivariate logistic Cox Regression.

	Univariate logistic regression		Multivariate logistic regression	
	P	HR (95% CI)	P	HR (95% CI)
Low output syndrome	<0.001	4.588 (2.570–8.189)	<0.001	4.301 (2.108–8.776)
Operation time	0.002	1.004 (1.001–1.006)		
Bypass time	0.032	1.005 (1.000–1.009)		
IABP implantation	0.026	1.948 (1.084–3.499)		
Red blood transfusion	0.012	1.000 (1.000–1.001)		
Vasopressin 24h max.	<0.001	1.156 (1.073–1.246)	0.027	1.108 (1.012–1.213)
Epinephrine 24h max.	0.005	5.112 (1.646–15.877)		
Lactate 24h max.	0.003	1.008 (1.003–1.014)		

CI indicates 95% confidence interval. HR indicates hazard ratio

**Fig 1:**
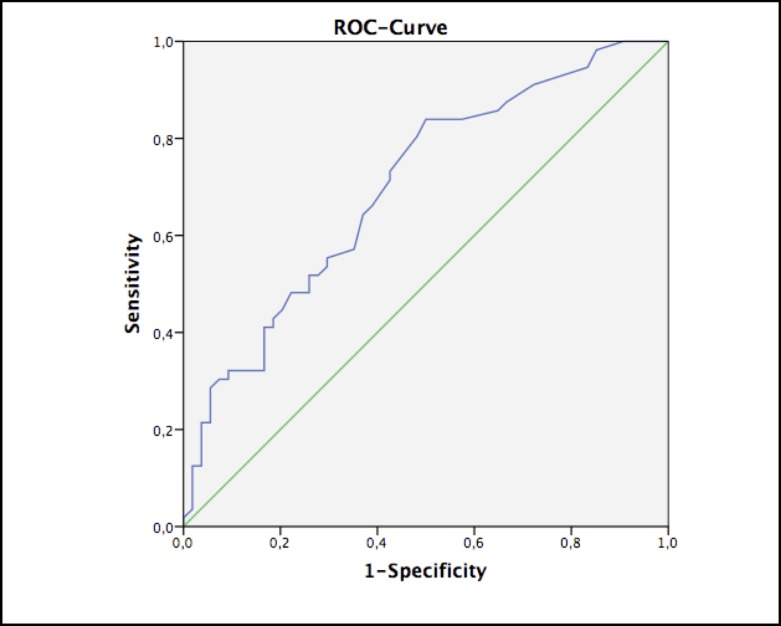
Receivers operating curve (ROC) for prediction power of lactate value with AUC of 0.7

For prognostic power analysis of lactate value within first 24h ROC analysis was asserted, which showed an area under the curve (AUC) of only 0.70. Therefore, the best prognostic cut-off value for lactate was 22.5mg/dl and above. This value has a sensitivity of 73.2% and specificity of only 57.4% (Fig 1). Further ROC testing revealed an AUC of 0.698 for maximum vasopressin dosage and 0.660 for maximum epinephrine dosage therapy within 1^st^ POD and was hence not further investigated.

## Comment

To our knowledge, this is the first study to conduct a matched-pair analysis to detect risk factors for the development of IC with emphasis on the early postoperative catecholamine therapy after cardiac surgery. In this single-center study of patients undergoing laparotomy and consecutive colectomy after initial cardiac surgery with the help of ECC the complication of ischemia of the large intestine resulted in an in-hospital mortality rate of 67%. Earlier reports described mortality rates between 30–100% in patients with mesenteric ischemia after CPB [1–4]. However, most of the other studies described gastrointestinal complications after cardiac surgery in general. Others compared colectomy of all causes to patients who were treated medically without surgical intervention [8, 9]. A comparison of medically treated patients with colonic complications after cardiac surgery is not eligible in our view. Such study would require a standardized screening method for each patient such as mesenteric angiography or abdominal x-ray, since bio-markers are not available yet. Mucosal damage markers, e.g. intestinal fatty acid-binding protein, seem to be not significantly predictive [10]. Demir et al. found positive effects of silymarin in a rat mesenteric ischemic-reperfusion model [11]. During the study period no prophylactic medication was treated at our institute besides proton-pump inhibitor and H2 blocking, but silymarin seems to be a possible option for clinical trials in the future.

The cause of ischemia of the large intestine is arguable, except for OMI which is induced by vascular obstruction weather by embolism or thrombosis, as seen in one patient in our cohort, who diseased on 10^th^ POD. Determining cardiopulmonary bypass as the major cause of colonic complications is not legit, especially if patients are diagnosed during the late postoperative course. However, CPB is known to cause systemic inflammatory response syndrome (SIRS) leading to barrier loss, which also occurs in the mesenteric mucosa. Besides decreased mesenteric blood flow, regional intestinal differences in perfusion accompanied by hypothermia and normothermia as well as hyper-reagibility to vasoconstrictors aggravate mucosal ischemia [12, 13]. Furthermore, perioperative factors such as anesthesiological medication and surgery itself may also contribute to mesenteric damage [14]. This damage results in the destruction of the mucosal layer leading to bacterial translocation into the blood with consecutive development of multi-organ failure [9]. Besides the pathophysiological factors that cause intraoperative mesenteric ischemia, several predictive risk factors have been described in retrospective studies. Ghosh et al. found duration of cross-clamp, use of significant inotropic support, intra-aortic balloon counterpulsation for low cardiac output, need for blood transfusions, triple vessel disease and peripheral vascular disease to predict mesenteric ischemia which occurred in 39 patients (0.07%) with a mortality of 64% compared to 5394 patients without ischemia [15]. Groesdonk et al. described in a prospective cohort among preoperative parameters renal insufficiency, diuretic therapy, and age over 70 years to be significant predictive factors for NOMI. The highest odds ratios for development of NOMI were need for intra-aortic balloon pump support and serum lactate concentrations >5 mmol/L postoperatively [16].

We included only three cardiac procedures to match the study group optimally and generate valid results. Both study arms showed almost no significant differences in several descriptive perioperative data proving the goodness of matching. No significant differences were found in rate of emergency operation, NYHA, PAD, COPD, nicotine, creatinine, dialyses, myocardial infarction or reduced LV function of which some have been reported to be predictive risk factors for in-hospital mortality in several other cohorts [1–4, 17, 18]. Univariate regression revealed bypass- and operation-time, but not cross-clamp time to be risk factors for the development of IC, which underlines CPB as a major risk factor, though multivariate analysis showed no significant influence. We found relatively high catecholamine doses and lactate values within the first POD in the complete cohort but surprisingly no significant differences between the two groups for vasopressor therapy with epinephrine or norepinephrine, but vasopressin. After CPB patients often require stimulation with catecholamines, phosphodiesterase III inhibitors and levosimendan, which can interfere with auto-regulation of the mesenteric blood flow resulting in hypoperfusion and vasospasm [19, 20]. Regression analysis defined vasopressin therapy as a risk factor for the development of IC. However, this finding is controversial. In our center, vasopressin is usually administered, if norepinephrine and/or epinephrine levels are excessive. Therefore, patients who already suffer from a massive low cardiac output syndrome or massive vasoplegic syndrome receive treatment with vasopressin. Thus, low output per se could have already led to irreversible damage to the intestine and resulted in splanchnic malperfusion. But malperfusion could have also been aggravated by vasopressin. On the contrary, Bomberg et al. recently claimed that vasopressin could lead to positive effects and survival improvement in patients suffering from NOMI after cardiac operations [21]. However, the study cohort was relatively small and further investigations are necessary to verify these findings. The same investigators found vasopressin to reduce blood flow in the superior mesenteric artery and in the rectosigmoidal mucosa, and adversatively improved distal jejunal microvascular blood flow, which underlines the ambiguity of the vasopressors’ effects [22].

Low cardiac output with an HR of 4.301 remains the biggest threat for colonic complications in our cohort. Though it remains unclear, if the syndrome is a consequence of complications due to CPB or anesthesiology or even preoperatively existing clinical limitations or part of the vicious circle in which IC leads to low cardiac output or vice versa. Nevertheless, if low cardiac output is diagnosed, intestinal complications, especially IC, must be thoroughly considered.

Elevated serum lactate is described as an early indicator for mesenteric ischemia [15]. In contrast, Hasan et al. did not find metabolic acidosis and elevated serum lactate to be necessarily elevated, even during extensive ischemia [23, 24]. The ambiguous findings of our ROC analysis regarding the predictive power of lactate, especially considering that our estimated cut-off value is clinically regarded as a tolerable postoperative lactate level, confirms, that lactate cannot solve the clinical problem of the early detection of IC. The overall ROC findings with borderline or inferior AUC values may be related to our cohort size and to the high discrepancy of patients in need of epinephrine or vasopressin during 1^st^ POD.

Tompeter et al. reported that early laparotomy after initial operation can be life-saving [25]. This may be a key point in the therapy of this extremely life-threatening complication. In our cohort patients underwent surgery on 10^th^ POD in average, which still led to a high mortality rate of 67%. An earlier aggressive surgical approach could be considered, however, conservative treatment also requires further investigation.

The significant postoperative differences in the descriptive analysis can be causally connected to multiple end-organ failure and prolonged ICU stay of the IC group with significantly higher bilirubin levels and need of transfusion, which are not a cause but a consequence of long-term intensive treatment.

Patients undergoing colectomy due to IC after primary cardiovascular operation with help of ECC have a substantial poor outcome as previously described. Vasopressin administration seems to elevate the risk of IC, especially if low cardiac output syndrome is diagnosed, in which’s presence IC should be considered at any time. Elevated lactate levels remain a possible predictive factor, with low statistical predication.
